# ISSVA Classification of Vascular Anomalies and Molecular Biology

**DOI:** 10.3390/ijms23042358

**Published:** 2022-02-21

**Authors:** Kayo Kunimoto, Yuki Yamamoto, Masatoshi Jinnin

**Affiliations:** Department of Dermatology, Wakayama Medical University Graduate School of Medicine, 811-1 Kimiidera, Wakayama 641-0012, Japan; k-jigen@wakayama-med.ac.jp (K.K.); yukiy@wakayama-med.ac.jp (Y.Y.)

**Keywords:** venous malformation, lymphatic malformation, infantile hemangioma

## Abstract

Vascular anomalies include various diseases, which are classified into two types according to the International Society for the Study of Vascular Anomalies (ISSVA) classification: vascular tumors with proliferative changes of endothelial cells, and vascular malformations primarily consisting of structural vascular abnormalities. The most recent ISSVA classifications, published in 2018, detail the causative genes involved in many lesions. Here, we summarize the latest findings on genetic abnormalities, with the presentation of the molecular pathology of vascular anomalies.

## 1. Introduction: ISSVA Classification

Vascular lesions had been habitually termed ‘hemangioma’ or ‘angioma’ in both Japan and Europe/the United States based on the impression that most anomalies are ‘tumors’. On the contrary, hemangioma simplex and cavernous hemangioma, for example, are actually morphological abnormalities of capillary blood vessels or veins, respectively, despite the disease name “hemangioma”. These diseases differ from tumors in a narrow sense, which refers to autonomous cell proliferation. In addition to such problems with disease naming and nomenclature, vascular lesions can occur at various ages and in various organs, meaning they may require treatment in various hospital departments, so improved common terms/language are essential for mutual understanding.

Under such circumstances, Mulliken and Glowacki described differences between infantile hemangioma with the proliferation of vascular endothelial cells (ECs) and vascular malformations characterized by the abnormal dilation of vessels without proliferation. Later, the first version of the International Society for the Study of Vascular Anomalies (ISSVA) classification was adopted at a workshop in Rome in 1996, and has been revised several times since. Initially, this was a simple classification of vascular anomalies into two groups (tumors and malformations), but new diseases/conditions and subtypes have since been included in subsequent revisions, and a large number of causative genes have been mentioned. The ISSVA classification is a basic and systematic classification of vascular anomalies with international acceptance, so its use infers the standardization of diagnosis and treatment [1].

We, therefore, review the latest findings on genetic abnormalities, with the presentation of the molecular pathology of vascular anomalies in relation to the most recent (2018) ISSVA classification.

## 2. Structure of the ISSVA Classification

In the most recent ISSVA classification, there are mutual links between several tables. In the first overview table (Table 1), vascular anomalies are primarily classified into two types: “vascular tumors”, which include proliferative changes of ECs, and “vascular malformations”, which are structural vascular abnormalities without EC proliferation. The former is subclassified into three types: benign (e.g., infantile hemangioma, congenital hemangioma, and tufted angioma (TA)), locally aggressive or borderline (e.g., kaposiform hemangioendothelioma (KHE), and Kaposi’s sarcoma), and malignant (e.g., angiosarcoma and epithelioid hemangioendothelioma).

Meanwhile, vascular malformations are subclassified into four types. The simple type is further subclassified based on the type of blood vessels with anomalies: capillary, lymphatic, venous, and arteriovenous malformations. In the combined type, two or more simple vascular malformations are found in one lesion. The malformation of major named vessels refers to abnormalities in the origin/course/number of major blood vessels that have anatomical names. Malformations associated with other anomalies include syndromes in which vascular malformations are complicated by symptoms other than vascular anomalies, including soft tissue or skeletal abnormalities, such as leg-length discrepancy and segmental hypertrophy (e.g., Klippel–Trenaunay syndrome and Sturge–Weber syndrome). In addition, some relatively rare diseases are presented as “provisionally unclassified vascular anomalies”.

Elsewhere, a description on the PIK3CA-related overgrowth spectrum (PROS) has been added as an appendix. PROS is now defined to include diseases with heterogeneous segmental overgrowth phenotypes due to the somatic activating mutations of PIK3CA (gene coding PI3K p110α subunit). PROS is stated to often be accompanied by various vascular malformations.

## 3. Gene Mutations and Molecular Biological Mechanisms in Vascular Anomalies

**Figure 1:**
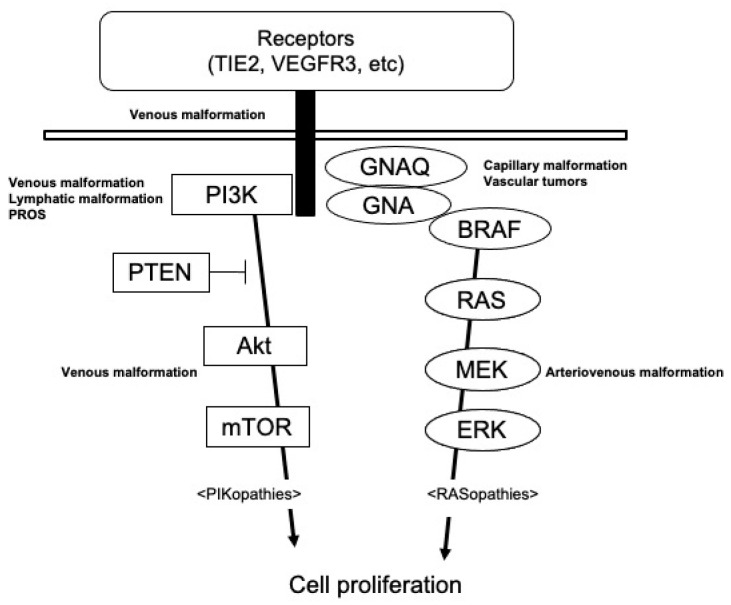
Diagram illustrating signaling pathways involved in the pathogenesis of vascular anomalies.

As described above, the identification of gene mutation(s) in each disease through the widespread use of next-generation sequencers is a clue to the understanding of the 2018 ISSVA classification. Causative genes for vascular anomalies are often found on molecules on the RAS/MEK/ERK pathway and PIK3CA/Akt/mTOR pathway (Figure 1). The RAS/MEK/ERK pathway, as the so-called RASopathy, mainly causes high-flow vascular malformations, including arteriovenous malformations and vascular tumors. On the other hand, the PIK3CA/Akt/mTOR pathway, as PIKopathy, induces slow-flow vascular malformations, such as venous or lymphatic malformations. The clinical characteristics and molecular biological mechanisms of each lesion are described below.

### 3.1. Venous Malformation

Venous malformation was previously known as “cavernous hemangioma”. It manifests as a soft subcutaneous mass with a normal to blue-purple coloring surface. Many cases are sporadic, while hereditary types are known as “familial mucocutaneous venous malformations”.

Among vascular anomalies, venous malformations are one of the diseases in which causative genes are primarily identified. A genetic analysis using blood samples from patients with familial mucocutaneous venous malformations initially identified the presence of heterozygous germline missense point mutations: a C-to-T nucleotide transition leading to an arginine-to-tryptophan substitution at position 849 in the kinase domain of Tie2 [2]. It was unclear at that time, however, why localized lesions occurred without involving all blood vessels, despite the presence of germline mutations in all cells of a patients’ body, and why the penetrance of the mutation was low.

**Figure 2:**
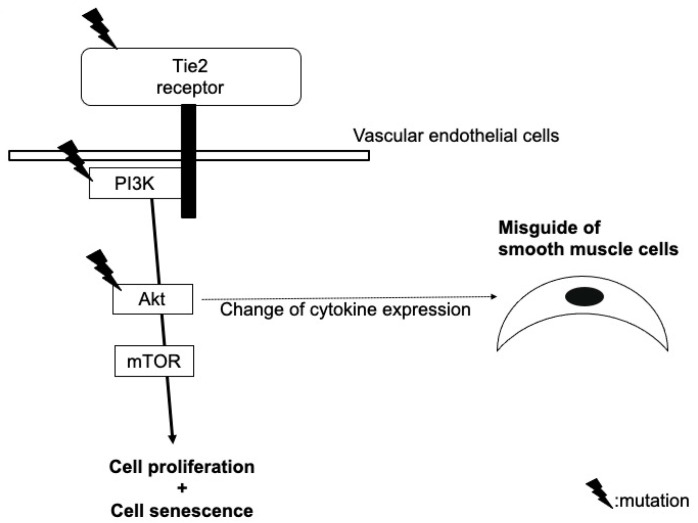
Our hypothetical model of the role of mutations in Tie2, PIK3CA, and Akt in the pathogenesis of venous malformation.

Other several somatic heterozygous mutations (e.g., p.L914F) were, thereafter, identified in a Tie2 gene allele, which was normal in blood DNA and in the lesional DNA of patients with familial venous malformations [3]. Such ‘second-hit’ mutations in lesional DNA explain the reasons for localized lesions with the noninvolvement of all blood vessels and the low penetrance of the mutation. Tie2 mutations are now known to also be found in patients with sporadic venous malformations, and it is recognized that some patients also have mutations in PIK3CA or Akt, which are downstream molecules of Tie2. The mechanism by which these gene mutations induce characteristic morphological abnormalities of veins still requires clarification, but there are several hypotheses (Figure 2).

For example, Tie2 is a receptor molecule that is specifically expressed on ECs; there should, therefore, be no abnormalities of vascular smooth muscle cells themselves in a venous malformation. However, Tie2 mutations induces autophosphorylation, not expression, and a dysregulated Tie2 signaling pathway due to the mutations in ECs may affect the expression of cytokines, such as angiopoietin-2 and PDGF [4], resulting in the misguiding of smooth muscle cells to the surroundings of blood vessels and leading to an abnormal venous dilation. A second hypothesis is that the gene mutations of the Tie2 signaling pathway induce the clustering, proliferation, or chemotaxis of ECs [5]. Simultaneously, mTOR at the downstream of this pathway induces cell senescence; this leads to morphological abnormalities, but not tumorigenesis [6]. Thirdly, changes in the angiogenic potential, blood vessel permeability of ECs, or arteriovenous identity loss induced by the Tie2 mutations may be involved in the abnormal dilation [4].

### 3.2. Glomuvenous Malformation

In the ISSVA classification, a glomuvenous malformation is classified into the category of venous malformations. Histologically, the lesions are due to the proliferation of immature smooth muscle cells (glomus cells). Sporadic cases can be painful and appear below the nails, while hereditary cases can manifest as multiple lesions.

Heterozygous germline mutations in the glomulin gene have been identified in the gene analysis of blood DNA in patients with familial glomuvenous malformations (e.g., the deletion mutation of 157delAAGAA and point mutation c.C108A), but with a low penetrance. An analysis of the lesional DNA in individual patients confirmed second-hit mutations of the glomulin gene (980delCAGAA) [7].

**Figure 3:**
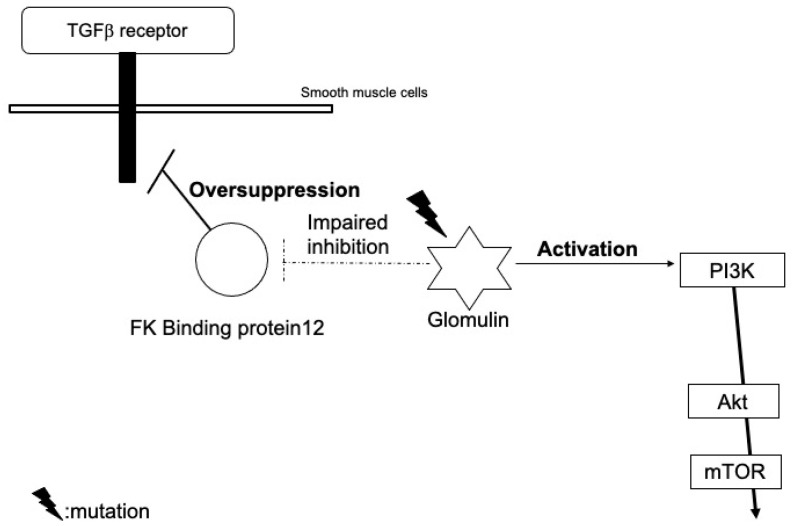
Our hypothetical model of the role of glomulin mutations in the pathogenesis of glomuvenous malformation.

This somatic mutation is not likely to affect the expression, but cause the loss-of-function of glomulin. Wild-type glomulin proteins bind to and inhibit the FK binding protein 12 (Figure 3). The FK binding protein also blocks TGF-β signals, but mutant glomulin cannot inhibit the FK binding protein, which leads to an excessive reduction in TGF-β signals [7]. Immature glomus cells may proliferate due to the impairment of the TGF-β-mediated smooth muscle cell differentiation.

Furthermore, they may also activate PI3K signals through interactions with the hepatocyte growth factor receptor, c-met [8].

### 3.3. Lymphatic Malformation

Lymphatic malformation is a congenital lymphatic dysplasia. It is classified into two types: the macrocystic type, in which 1–3 large cystic lesions are present, and the microcystic type, in which small cystic lesions are aggregated. In addition to an abnormal expression of the molecules involved in lymphangiogenesis, such as the vascular endothelial growth factor (VEGF)-C and VEGF receptor type3 (VEGFR3), PIK3CA mutations have been detected in lymphatic ECs of the lesions [4]. Similar to epithelial cancers, the most common PIK3CA mutations found in lymphatic malformation are activating mutations in the helical (p.E542K) and kinase (p.H1047R and p.H1047L) domains [9]. p.E109del, p.C420R, p.E545K, p.Q546K, and p.H1047L have also been reported [10].

In mice, forced changes in p110α activity led to embryonic lethality due to the incomplete development of blood vessels; the PIK3CA signal may, therefore, be a pathway essential for vascular development [4]. PIK3CA mutations in lymphatic ECs may stimulate the expression of vegf-c or vegfr3, induce the binding of pik3ca to the cellular membrane, or increase cell proliferation, chemotaxis, and angiogenesis through the activation of downstream akt/mtor [8]. similar mechanisms to venous malformation may exist in the process of the abnormal lymphatic dilation (e.g., changes in chemotaxis, proliferation, or angiogenic potential of ecs and misguide of vascular smooth muscle cells) [4].

The early embryonic activation of p110α was indicated in a mouse model study to, predominantly, lead to the formation of lesions that recapitulated features of human macrocystic lymphatic malformation, whereas its activation during late embryonic or neonatal development resulted in microcystic lesions. Accordingly, the cell circumstances/environments at the timing of gene mutation occurrence may explain differences in the clinical features of lymphatic malformations.

### 3.4. Arteriovenous Malformation

Arteriovenous malformation is defined as a congenital abnormal connection of arteries and veins, which disrupts normal blood flow and oxygen circulation. According to the Schobinger Staging system, clinical manifestation varies from cutaneous warm, pink-blue shunting to ulceration, bleeding, and cardiac failure.

Mutations in the genes of the RAS pathway, including KRAS (e.g., p.G12D and p.G12V), MAP2K1 (e.g., p.F53L, p.Q56P, p.K57N, p.Q58del, and p.D67Y), and BRAF (e.g., p.V600E), have been detected [11,12,13]. Although the influence of these mutations on the expression and activation of RAS pathway molecules is not fully understood, downstream MEK/ERK signals have been found to be activated in the lesion [14]. In addition, morphological changes in ECs, an increase in sprouting behavior, the enlargement of the vessel lumen, and abnormal connections between arteries and veins without cell proliferation are induced by RAS activation; gene mutations in the RAS pathway may be, therefore, strongly involved in the pathogenesis [15].

### 3.5. Klippel–Trenaunay Syndrome

Klippel–Trenaunay–Weber syndrome was previously defined as a triad of hemangioma simplex, varicose veins, and bony/soft tissue hypertrophy involving an extremity. It is currently classified into two distinct diseases; Parkes Weber syndrome is defined as vascular malformations involving high-flow arterial components (arteriovenous fistulae) in addition to affected-limb hypertrophy, whereas Klippel–Trenaunay syndrome is combination of slow-flow vascular malformations (capillary, lymphatic, and venous components) accompanied with limb hypertrophy.

PIK3CA was identified as a causative gene of Klippel–Trenaunay syndrome, and the disease has been regarded as one of PROS. A recent study identified any one of five mutations (p.C420R, p.E542K, p.E545K, p.H1047R and p.H1047L) in 20 of 21 patients [16].

PIK3CA mutations, on the other hand, are also detected in lesions of venous or lymphatic malformations. Germline PIK3CA mutations, as described above, lead to embryonic lethality. Venous or lymphatic malformations are usually localized, so mutations are thought to occur late during development, affecting a single clone of ECs [4]. On the other hand, mosaic mutations in the early embryonic phase may result in PROS. These PIK3CA mutations have also been detected in adult epithelial tumors, such as breast cancers or colon cancers, but other gene mutations are also present in many cases [4]. However, PIK3CA mutations alone do not induce overgrowth in mice models [5], so there is also a possibility that environmental factors, other gene mutations, or mutations in other cells, such as fibroblasts, may be required for the formation of overgrowth lesions [4,5].

### 3.6. Sturge–Weber Syndrome

This syndrome was originally regarded as neurocutaneous disease, which involves a facial hemangioma simplex reaching the first branch of the trigeminal nerve, ophthalmologic abnormalities (especially congenital glaucoma), and neurologic signs (seizure, mental retardation, hemiparesis).

Mosaic gene mutations of GNAQ (e.g., p.R183Q) or GNA11 (p.R183C, p.R183H, p.Q209L, and p.Q209P) in ECs were recently detected [17,18,19]. They did not change the protein expression, and are thought to activate downstream MEK/ERK pathways, but still require clarification of the detailed mechanism. Furthermore, the mechanism by which the mutated GNAQ/GNA11 genes induce capillary malformations is also still unknown, but there are several hypotheses. First, GNAQ or GNA11 protein may be related to EC sensing of shear stress imposed by blood flow, and their mutants may impair the ability of EC to distinguish between laminar and disturbed flow [20]. A second hypothesis is that GNAQ gene may activate the PIK3/Akt pathway in addition to the MEK/ERK pathway, and PIK3CA mutations have also been found in Sturge–Weber syndrome [18]. Abnormal capillary dilation may occur via the same mechanism as indicated for venous or lymphatic malformations through PIK3CA/Akt/mTOR. A third hypothesis is that EC differentiation may be impaired by the mutations, leading to progressive dilatation of immature venule-like vasculatures [18]. Recently, the gene mutations of GNAQ and GNA11 have also been detected even in the lesions of sporadic capillary malformation.

### 3.7. Infantile Hemangioma

Concerning vascular tumors, causative genes have not been sufficiently identified in comparison to vascular malformations. For example, infantile hemangioma is a benign vascular tumor caused by the uncontrolled proliferation of vascular ECs. Causative genes have not been described in the ISSVA classification 2018, but cultured infantile hemangioma-derived ECs (hemECs) have been found to have an increased proliferation and migration activity [21]. Furthermore, they are clonal based on analyses of X-chromosome inactivation, while non-ECs from the lesions did not exhibit clonality [22]. Infantile hemangioma has, therefore, been thought to be caused by an “intrinsic abnormal activation” of ECs that leads to local clonal expansion, rather than a secondary response of ECs to external factors.

The following hypothesis was first proposed as a possible molecular mechanism: hemangiomas are clonal expansions of cells that have originated from embolized placental cells or bone marrow cells. For example, the embolism of maternal placenta-derived cells may occur in the fetal skin, leading to localized proliferation. This is based on the finding that the expression pattern of cellular markers (e.g, GLUT-1, merosin, FcRII, Lewis Y antigen, type 3 iodothyronine deiodinase, indoleamine 2,3-deoxygenase, and IGF2) and transcriptomes in infantile hemangioma tissue differ from that of ECs in the surrounding skin, but resemble those of ECs lining fetal microvessels in the human placenta [23]. This hypothesis is also supported by the evidence that chorionic villous sampling, which causes trauma to the placenta and may induce cellular transfer between maternal and fetal circulations, increases the incidence of the tumor. In addition, the high incidence of infantile hemangioma in preterm babies may also be associated with placental complications that lead to premature birth. There is no direct evidence for this, however, such as the detection of mother-derived XX chromosome cells in the lesions of infantile hemangioma in boys [24].

According to another hypothesis, tumor cells of infantile hemangioma might be derived from undifferentiated stem cells or progenitor cells (e.g., dormant angioblasts or cells recruited to the lesions from a reservoir of stem/progenitor cells). Khan et al. identified infantile hemangioma-derived endothelial progenitor cells (hemEPCs) by their mRNA expression patterns or their response to endostatin, and indicated that hemEPCs and hemECs share common properties with cord blood EPCs [25]. In addition, Kleinman reported an elevation of the level of circulating EPCs in babies with infantile hemangioma, and that they may be recruited into tumors at the proliferating stage, leading to tumor formation [26]. Furthermore, mesenchymal stem cells derived from infantile hemangioma (hemMSCs) at the proliferating stage exhibited higher adipogenic activity than those from lesions at the involuting stage and from normal skin, supporting the hypothesis that MSCs reside in the tumor and are the source of fibrofatty tissues seen in the involuted phase [27]. Infantile hemangioma-derived stem cells (hemSCs) express CD90, a mesenchymal cell marker, which is one of the genes upregulated in proliferating lesions compared to involuting lesions [28,29]. Normal human dermal microvascular ECs (HDMECs) required mesenchymal supporting cells to form vessels in subcutaneous implants in immunodeficient mice [30], whereas the implantation of hemSCs alone could form functional vessels exhibiting an infantile hemangioma-like phenotype with a high GLUT-1 expression and could differentiate into adipocytes [28]. Other cell types, including hemECs, hemEPCs, cord blood EPCs, normal human fibroblasts, and bone marrow-derived mesenchymal stem cells, did not form vessels in the same mouse model. Accordingly, hemSC may mainly represent progenitor cells for infantile hemangioma.

On the other hand, we have also demonstrated that hemECs maintain activated VEGF signaling pathways in vitro [31]. VEGF receptor type 2 (VEGFR2) and downstream signaling molecules such as ERK and Akt were constitutively activated in cultured hemECs, resulting in the upregulated proliferation and migration in the absence of exogenous VEGF [31].

In addition, as the cause of VEGFR2 activation in hemECs, we found the downregulation of VEGF receptor type 1 (VEGFR1), resulting in an increase in VEGF that binds to VEGFR2. Interestingly, VEGFR1-null mice died at E8.5 to E9.0 due to overgrowth of ECs and the disorganization of blood vessels, both of which are characteristic of infantile hemangioma [32,33]. An abnormality in pathways that control the VEGFR1 expression may, therefore, play a significant role in the pathogenesis of infantile hemangioma.

As the mechanism of VEGFR1 downregulation, we identified a putative binding site of the nuclear factor in activated T cells (NFAT) on a region of the VEGFR1 promoter. NFAT is known to be activated by the Ca^++^/calmodulin-dependent phosphatase calcineurin, and a low NFATc2 activation and low basal levels of cytoplasmic Ca^++^ were found in hemECs. In addition, the expression of active β1 integrin determined by the HUTS-21 antibody was reduced in hemECs compared with in HDMECs. A link between integrins and Ca^++^ signaling has been well established, and the inactivation of the β1 integrin may cause the suppression of the Ca^++^-NFAT-VEGFR1 pathway in hemECs. Consistently, the activation of the β1 integrin by its stimulatory antibody in hemECs could reduce VEGFR2 phosphorylation, induce the binding of NFATc2 to the VEGFR1 promoter, and stimulate the expression of VEGFR1.

We also found germline heterozygous amino acid substitutions in tumor endothelial marker-8 (TEM8) and VEGFR2 in a portion of hemECs: a G-to-A transition replaces alanine by threonine in the transmembrane domain of the integrin-like molecule TEM8 (p.A326T). On the other hand, a T-to-C transition changes cysteine into arginine at position 482 in the Ig-like domain V of the VEGFR2 extracellular region.

TEM8 is also known as Anthrax toxin receptor 1, and is an integrin-like receptor expressed in ECs [34]. The overexpression of wild-type TEM8 in hemECs with mutated TEM8 induced the amount of activated β1 integrin, stimulated the association between NFATc2 and the Flt-1 promoter, and upregulated VEGFR1 expression. In turn, the overexpression of mutant TEM8 in HDMECs induced an infantile hemangioma-like phenotype. The overexpression of wild-type VEGFR2 in hemECs with mutated VEGFR2 also normalized the infantile hemangioma-like phenotype.

Immunoprecipitation assays showed that VEGFR2 could form complexes with β1 integrin and TEM8 in hemECs, and that mutations in VEGFR2 and TEM8 resulted in an increased interaction among the three proteins. An increased complex formation among the three molecules and the subsequent inactivation of the β1 integrin-NFATc2-VEGFR1 pathway are features that are common to all hemECs that have been tested, even to hemECs in which we did not yet find mutations. We speculate that hemangioma formation in cases where we did not find gene mutations may be associated with mutations in genes encoding other pathway components, perhaps other cell surface or cytoplasmic proteins that interact with integrins/TEM8/VEGFR2, components of the Ca^++^-NFAT-VEGFR1 pathway, or downstream targets of VEGFR2.

However, because hemECs exhibit clonality, the germline mutations in TEM8 and VEGFR2 must be associated with a secondary somatic event to trigger the clonal expansion of tumor cells within the hemangioma lesions [22]. We, therefore, conclude that the changes to the TEM8 and VEGFR2 amino acid sequence represent risk factor mutations for infantile hemangioma, similar to familial cases of venous malformation or glomuvenous malformations as described above. Given their emergence after birth and the age-dependent involution of infantile hemangioma, we also hypothesized that physiological events, including perinatal hypoxia or mechanical stress, during delivery would be a trigger of hemangioma formation in infants with germline mutations with TEM8 and VEGFR2.

To prove the hypothesis, we tried to plot the localization of 104 infantile hemangioma on the head and face as well as capillary malformations. As a result, the lesions of infantile hemangioma in the jaw or chin areas were significantly less common than other areas. This tendency was not found in 40 patients with capillary malformation. Mechanical stress to the jaw or chin areas may be lesser than in other areas in normal cephalic delivery, so these data may indicate the contribution of mechanical stress as a trigger of infantile hemangioma, not capillary malformation [35].

On the other hand, based on above hypothesis, we also tried to confirm that head and neck lesions were more frequent in the group in which infantile hemangioma appeared after birth compared with the patients in which it was present at birth [36]. A slight such tendency was observed, but the difference was not statistically significant. Meanwhile, we unexpectedly found a statistically significant increase in the frequency of multiple lesions in infantile hemangioma after birth compared with those present at birth. This may indicate there may be different triggers between the two groups. In other words, infantile hemangioma present at birth are likely caused by a local trigger, while lesions appearing after birth may be induced by systemic factors in addition to local triggers, such as cytokines related to systemic neovascularization after birth.

### 3.8. Tufted Angioma and Kaposiform Hemangioendothelioma

TA is a relatively rare and benign vascular tumor of infancy clinically presenting as violaceous, indurated, or nodular plaques with pain, focal hyperhidrosis, and hypertrichosis. Histopathologically, the tumor shows confluent lobules or “cannonballs” of spindled ECs with slitlike lumina embedded in a fibrotic background [37]. Some lesions show spontaneous regression, but the underlying pathobiology of TA is poorly understood.

On the other hand, KHE, first reported by Zukerberg et al. in 1993, is characterized by a locally aggressive/borderline malignant behavior and morphological features similar to Kaposi sarcoma. TA is currently thought to be the dermal counterpart of KHE with a benign clinical course due to the superficial origin.

Kasabach–Merritt syndrome (KMS), a consumptive coagulopathy characterized by profound thrombocytopenia, hypofibrinogenemia, and microangiopathic anemia with up to 24 percent mortality [37,38], may affect approximately 70% of all patients with KHE and approximately 10% of patients with TA [39].

Although the etiologies of TA, KHE, and KMP are poorly understood, TA and KHE mainly occur in early childhood, suggesting the involvement of gene mutations in their pathogenesis. Lim et al. found *GNA14* mutations c.614A>T (p.Q205L) in these lesions [40]. However, these mutations are not specific to TA and KHE, and they were also detected in other rare vascular tumors, which are potential differential diagnoses [37,38]. Ten Broek et al. analyzed the epigenetic genomewide methylation profile, and demonstrated that KHE and TA share a common origin without a relationship to vascular malformation [41].

## 4. Future Issues in Molecular Biologics of Vascular Anomalies

In vascular anomalies, although causative genes in each lesion have been identified, most mechanisms are still unknown, as described above. In particular, there are no gene mutations in all ECs at each lesion. For example, the frequency of alleles with mutations in the lesional tissue is, approximately, 10% in patients with lymphatic malformations or with Sturge–Weber syndrome. The mechanism by which such a small population of cells with mutated genes contribute to the formation of each lesion still requires clarification [20,42]. In addition, the positions of mutated nucleotides differ even in the same causative gene of each disease, so respective mutations may have different mechanisms.

PROS caused by PIK3CA mutations includes several diseases such as Klippel–Trenaunay syndrome, CLOVES syndrome, and megalencephaly-capillary malformation-polymicrogyria (MCAP). However, the reason for clinical features markedly differing among PROS is also unclear. Whether they depend on the positions of a mutated nucleotide of PIK3CA, the timing of occurring mutations, or cell types in which mutations occur still requires clarification.

## 5. Future Perspective of Novel Therapies

The potential usefulness of molecular targeting drugs for vascular anomalies was indicated, consisting of hypotheses on the pathogeneses of each disease, as described above. In the future, treatment with biologics may eventually become available (Table 2), as demonstrated for inflammatory skin diseases or malignant tumors.

In particular, Venot et al. showed that PI3K inhibitors were effective for PROS-associated vascular anomalies and tissue overgrowth [43]. Currently, sirolimus (rapamycin), an mTOR inhibitor, is one of the most promising drugs for various vascular anomalies, such as venous or lymphatic malformations [44]. A clinical trial of oral sirolimus for lymphatic malformations was conducted in Japan, and based on the favorable results of the trial, the drug was approved in 2021.

Numerous investigations have also reported the effect of sirolimus on KMP induced in TA and KHE, and mTOR has been implicated in the development of KMP, a fatal complication. Furthermore, lesions of TA and KHE themselves also shrink by oral sirolimus [45,46], indicating that the involvement of mTOR in the pathogenesis of TA and KHE, and that overlapping pathways with vascular malformations are activated in these lesions.

Meanwhile, mTOR inhibitors are considered to be ineffective for capillary malformations with the GNAQ mutation. However, rapamycin can enhance the efficacy of pulsed dye laser by suppressing neoangiogenesis after laser treatment [47]. We are currently conducting a phase II clinical trial of sirolimus gel for several vascular anomalies (venous malformation, lymphatic malformation, TA, and KHE) for the first time in the world. These attempts may broaden treatment options for intractable vascular anomalies.

## 6. Conclusions: Molecular Aspect

As shown in the summative table (Table 3), in venous malformation, gene mutations in Tie2/PIK3CA/Akt signaling pathway molecules in ECs may affect cytokine expression, resulting in the misguiding of smooth muscle cells to the surrounding blood vessels and leading to abnormal venous dilation. These mutations may also induce both proliferation and senescence of ECs, which leads to morphological abnormalities.

In glomuvenous malformation, mutated glomulin proteins may activate PI3K signals through interactions with c-met. Furthermore, they may inhibit TGF-β-mediated smooth muscle cell differentiation and induce the proliferation of so-called glomus cells.

PIK3CA mutations in lymphatic malformation may stimulate the expression of VEGF-C or VEGFR3, induce the binding of PIK3CA to the cellular membrane, or increase cell proliferation, chemotaxis, and angiogenesis through the activation of downstream Akt/mTOR. Similar mechanisms to venous malformation may exist in the process of the abnormal lymphatic dilation.

RAS pathway mutations in arteriovenous malformation may activate MEK/ERK signals. In addition, morphological changes in ECs, an increase in sprouting behavior, the enlargement of the vessel lumen, and abnormal connections between arteries and veins without cell proliferation may be induced by RAS activation.

PIK3CA was identified as a causative gene of Klippel–Trenaunay syndrome, and mosaic mutations in the early embryonic phase may result in the development of this disease.

GNAQ or GNA11 mutations in capillary malformation may impair the ability of ECs to distinguish between a laminar and disturbed flow. A mutated GNAQ may also activate the PIK3/Akt pathway, and an abnormal capillary dilation may occur via the same mechanism as indicated for venous or lymphatic malformations.

Mutations in VEGFR2 and TEM8 in infantile hemangioma resulted in an increased interaction among VEGFR2, TEM8, and integrin. The subsequent inactivation of the integrin/NFATc2/VEGFR1 pathway may cause VEGFR2 phosphorylation and endothelial activation.

## Figures and Tables

**Table 1:** Overview table of ISSVA classification 2018, modified Table in Ref. [1].

Vascular Anomalies				
Vascular Tumors	Vascular Malformations			
Benign	***Simple***	***Combined***	***Of Major Named Vessels***	***Associated with Other Anomalies***
	Capillary malformations	defined as two or more vascular malformations found in one lesion	abnormalities in the origin/course/number of major blood vessels that have anatomical names	syndromes in which vascular malformations are complicated by symptoms other than vascular anomalies
Locally aggressive or Borderline	Lymphatic malformations			
	Venous malformations			
Malignant	Arteriovenous malformations *			
	Arteriovenous fistula *			

* High-flow lesions.

**Table 2:** Candidates of molecular targeting drugs for vascular anomalies.

Diseases	Target	Drug
	VEGF receptor	Pazopanib
PROS	PI3K	Alpelisib
PROS	Akt	Miransertib
Venous malformation Lymphatic malformation	mTOR	Rapamycin
Arteriovenous malformation	BRAF	Vemurafenib
Arteriovenous malformation	MEK	Trametinib

**Table 3:** Molecular aspect of vascular anomalies.

Diseases	Causative Genes	Possible Function
Venous malformation	*TIE2, PIK3CA, Akt*	Affect cytokine expression, resulting in misguiding of smooth muscle cells to the surroundings of blood vessels and leading to abnormal venous dilation Induce both proliferation and senescence of endothelial cells, which leads to morphological abnormalities
Glomuvenous malformation	*Glomulin*	Inhibit TGF-β-mediated smooth muscle cell differentiation and induce the proliferation of immature glomus cells Activate PI3K signals through interactions with c-met
Lymphatic malformation	*PIK3CA*	Stimulate cytokine expression Induce the binding of PIK3CA to the cellular membrane, or increase endothelial cell proliferation, chemotaxis, and angiogenesis
Arteriovenous malformation	*RAS*	Induce morphological changes in endothelial cells Induce sprouting behavior, enlargement of the vessel lumen, and abnormal connections between arteries and veins
Klippel-Trenaunay syndrome	*PIK3CA*	Mosaic mutations in the early embryonic phase cause segmental hypergrowth
Capillary malformation Sturge-Weber syndrome	*GNAQ, GNA11*	Impair the ability of endothelial cells to distinguish between laminar and disturbed flow Activate the PIK3/Akt pathway
Infantile hemangioma	*VEGFR2* *TEM8, etc.*	Increase the interaction among VEGFR2, TEM8 and integrin Subsequent inactivation of the integrin-NFATc2-VEGFR1 pathway cause VEGFR2 phosphorylation and endothelial activation
Tufted angioma Kaposiform hemangioendothelioma	*GNA14*	?

## Abbreviations

ECs	endothelial cells
HDMEC	human dermal microvascular ECs
hemECs	infantile hemangioma-derived ECs
hemEPCs	infantile hemangioma-derived endothelial progenitor cells
hemMSCs	infantile hemangioma-derived mesenchymal stem cells
hemSCs	infantile hemangioma-derived stem cells
ISSVA	International Society for the Study of Vascular Anomalies
KHE	kaposiform hemangioendothelioma
KMS	Kasabach-Merritt syndrome
MCAP	megalencephaly-capillary malformation-polymicrogyria
NFAT	nuclear factor in activated T cells
PROS	PIK3CA-related overgrowth spectrum
TA	tufted angioma
TEM8	tumor endothelial marker-8
VEGF	vascular endothelial growth factor
VEGFR1	VEGF receptor type 1
VEGFR2	VEGF receptor type2
VEGFR3	VEGF receptor type3
